# 3D-printed glenoid implant reconstruction, after partial scapulectomy for malignant tumors: a case series

**DOI:** 10.1007/s00590-024-03839-4

**Published:** 2024-01-27

**Authors:** Ioannis G. Trikoupis, Ioannis I. Mavrodontis, Dimitrios V. Papadopoulos, Stavros D. Goumenos, Dimitrios A. Georgoulis, Panagiotis Gavriil, Dimitra Melissaridou, Olga D. Savvidou, Vasileios A. Kontogeorgakos, Panayiotis J. Papagelopoulos

**Affiliations:** 1 First Department of Orthopedic Surgery and Traumatology, National and Kapodistrian University of Athens, School of Medicine, “ATTIKON” University General Hospital, Rimini 1, Chaidari, 12462 Athens, Greece; 2 Second Department of Orthopedics, National and Kapodistrian University of Athens, ‘Agia Olga’ Hospital, Th. Konstantopoulou 3-5, Nea Ionia, 14233 Athens, Greece

**Keywords:** 3D-printed implants, Partial scapulectomy, Glenoid tumor

## Abstract

**Purpose:**

Glenoid tumors are extremely rare, and reconstruction remains very challenging. The aim of this study is to present the clinical and functional outcomes, of a new glenoid reconstruction method using 3-dimensional-printed implant.

**Methods:**

Four patients with primary glenoid tumors underwent reconstruction using 3-dimensional-printed glenoid implant linked with reverse shoulder arthroplasty. We retrospectively reviewed the clinical and functional outcome, using MSTS and DASH score, as well as complications’ rate.

**Results:**

Wide excision was achieved in all patients. No local recurrence or distant metastasis was diagnosed at the follow-up period. The mean MSTS score was 80.5%, and DASH score was 15.2%. According to Hendersons’ classification, there were no postoperative complications.

**Conclusion:**

The use of 3-dimensional-printed implants, can be a very reliable solution with satisfying clinical and functional outcomes for reconstruction, in patients with musculoskeletal malignancies of the glenoid.

*Level of evidence* IV Treatment Study.

## Introduction

Although shoulder girdle area is the third most common anatomic location for musculoskeletal malignancies, scapular bone and soft tissue tumors are extremely rare [1]. The most common histopathological type of scapular malignancies include chondrosarcoma in adults, followed by Ewing sarcoma in children and young adolescents [2–5].

The development of endoprosthetic implants for the reconstruction of the resected bone tumors offered a reliable alternative to interscapular-thoracic resection (Tikhoff–Linberg operation) following total scapulectomy, providing better functional outcomes of these patients [6]. Recent advances in prostheses design offered an individualized strategy for every patient, thus nowadays endoprosthetic reconstruction is considered the gold standard for the surgical management of scapular tumors [1, 4, 7, 8].

However, surgical treatment of glenoid tumors remains controversial. Although partial scapulectomy and reconstruction using allografts or allograft-prosthetic composites is the mainstay for the management of glenoid tumors, the clinical results are suboptimal [5, 7, 9, 10]. Alone soft tissue reconstruction is another option for certain patients with preservation of the glenoid [11, 12]. 3D-printed implants have been widely used in orthopedic oncology over the past decade [13]. These implants are designed to reconstruct complex bone deficits in areas, such as the talus or calcaneus, the forearm, and the pelvic ring bones. Although larger studied are needed, the functional outcomes of these implants are promising [13–18].

The purpose of this study is to evaluate the short-term clinical and functional outcomes of enbloc resection and reconstruction using 3D-printed implant for the management of localized primary glenoid tumors.

## Patients and methods

A retrospective observational study of prospectively collected data was performed between 2018 and 2021, including patients who underwent partial scapulectomy and reconstruction using 3D-printed implant for the treatment of localized glenoid tumors. All patients had extensive glenoid resection, including the glenoid fossa and the neck of the scapula. The study received approval by the Institutional Board Review, while an informed consent was obtained from all patients. The minimum follow-up period is 12 months.

**Fig. 1:**
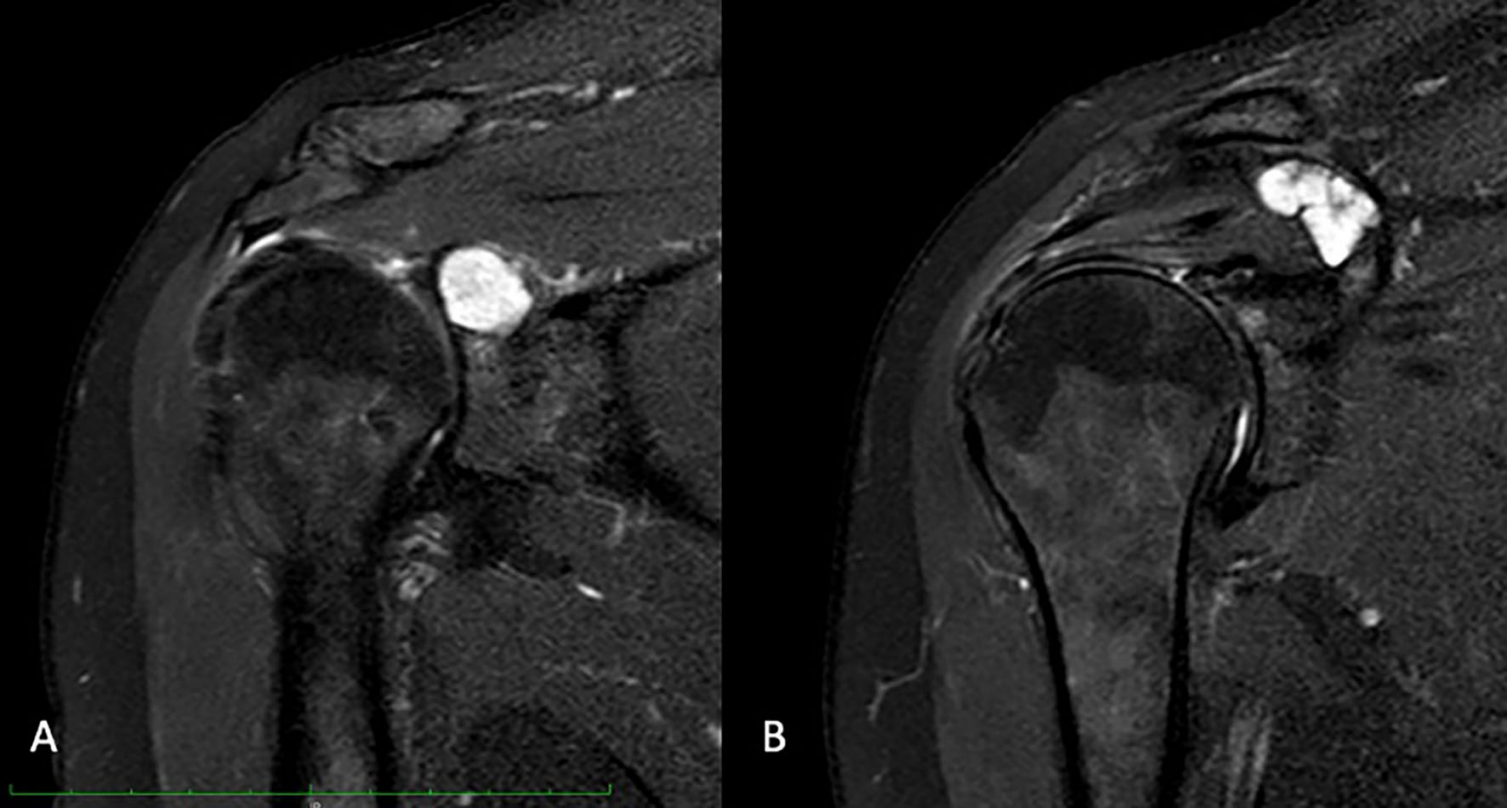
Preoperative T2 signal MRI of 56-year-old patient with a recurrent Grade 2 Chondrosarcoma of glenoid (**A**) and coracoid process (**B**)

**Fig. 2:**
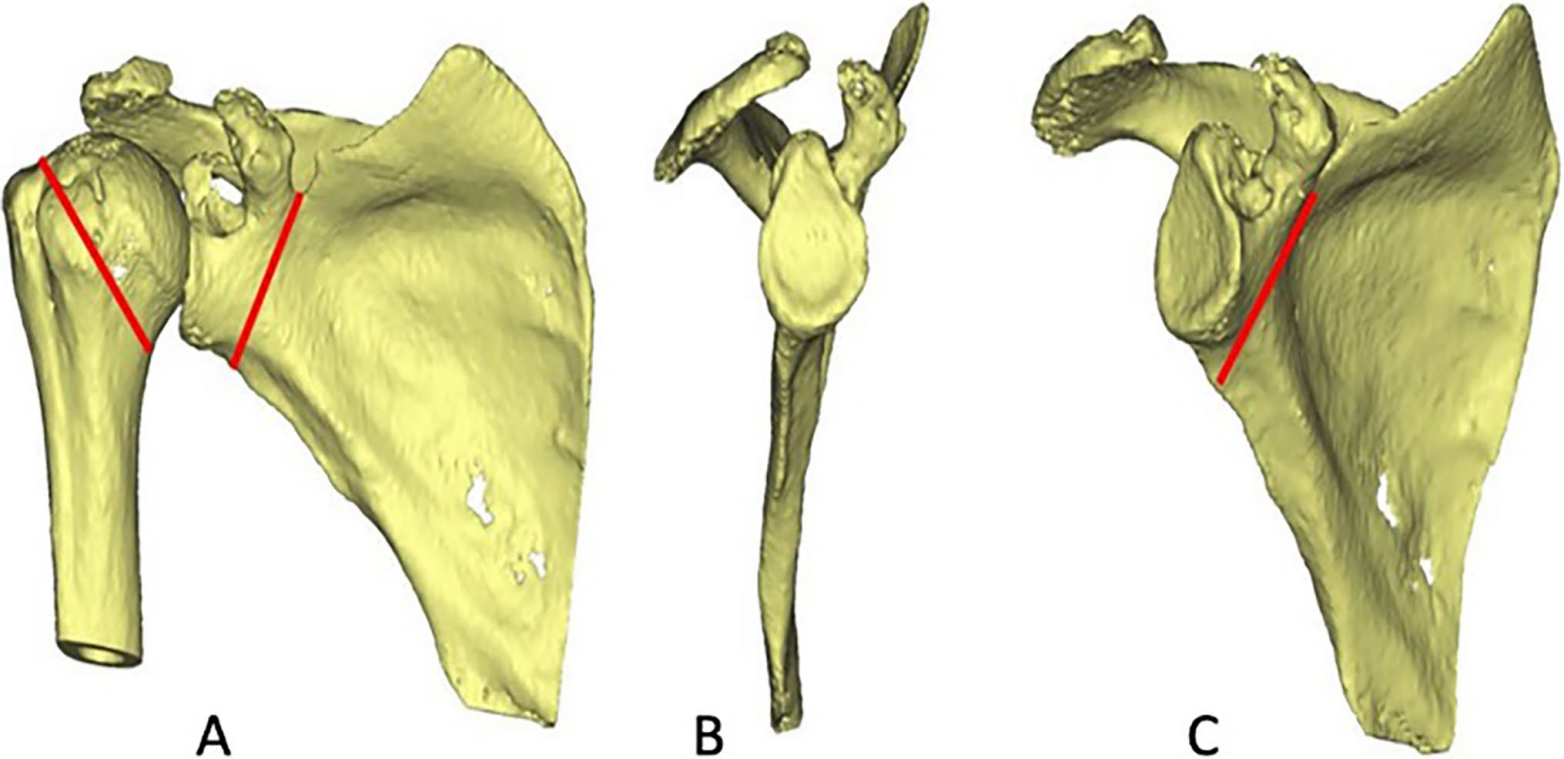
3D-reconstruction of the preoperative CT-scan of the right shoulder. Osteotomy planning in anterior (**A**), coronal (**B**) and posterior view (**C**) of the scapula

**Fig. 3:**
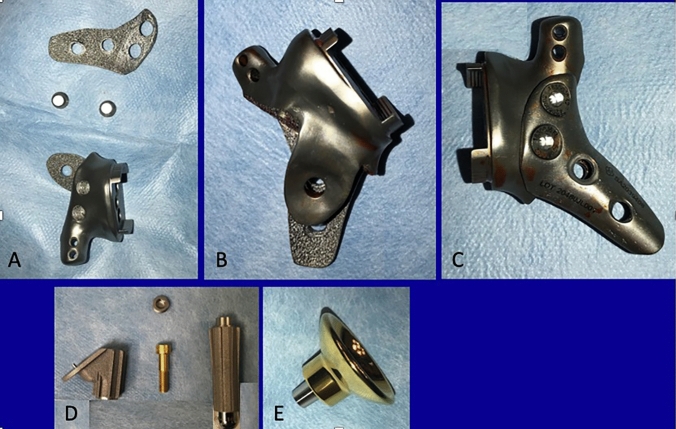
Final implant design. Modular fixation plate (**A**). Posterior (**B**) and anterior (**C**) view of the implant after plate assembling. Proximal humerus implants, humeral stem (**D**) and inverse cup (**E**)

**Fig. 4:**
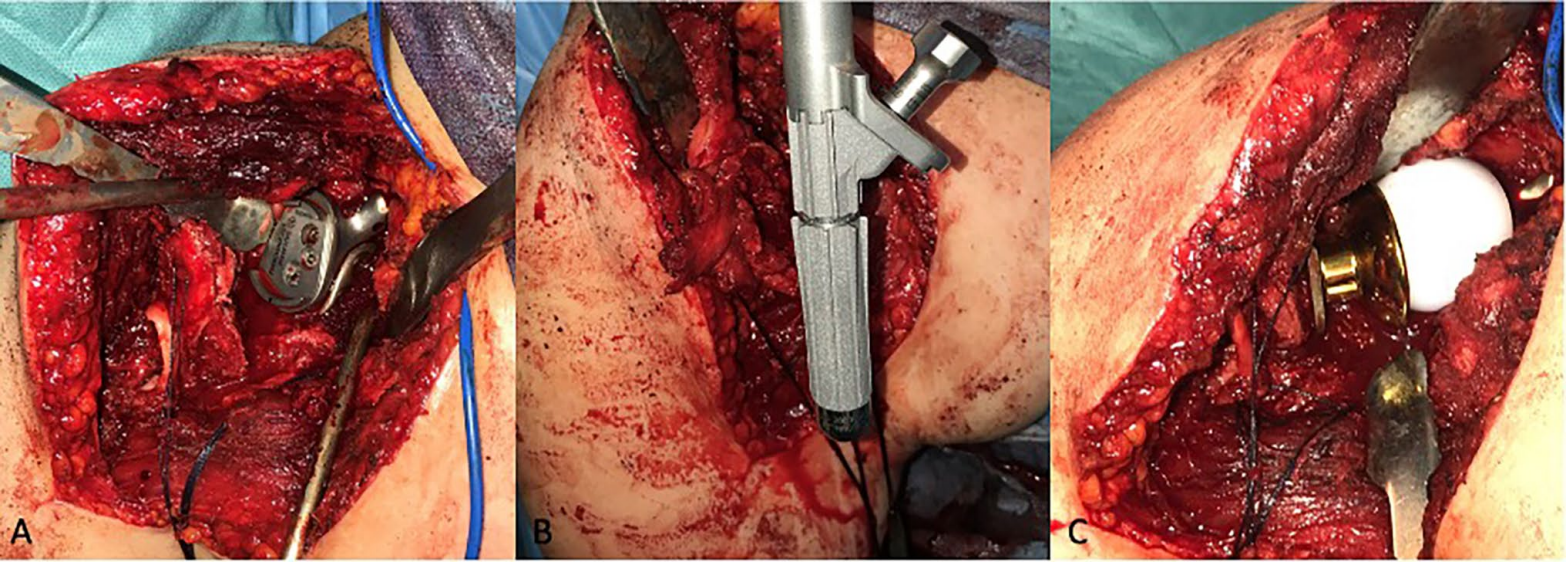
Custom made glenoid implant in place after tumor resection (**A**), proximal humerus stem insertion in the humeral canal (**B**) and the final reduction of the humerus to the glenosphere (**C**)

All patients underwent preoperative CT-guided needle biopsy and histopathological confirmation of the lesion. Imaging evaluation included CT and MRI of the shoulder for tumor staging, and the treatment strategy was discussed and planned in accordance with the multidisciplinary tumor board of the hospital. Four patients were diagnosed with chondrosarcoma. In one patient with chondrosarcoma, the tumor was a local recurrence following curettage and cementation, and the recurrence occurred two years postoperatively. For the preoperative planning, high-resolution CT scan and MRI were performed [19] (Figs. 1, 2). According to the Malawer surgical classification system for shoulder girdle resections, one patient underwent extraarticular Type V resection and the remaining 3, intraarticular Type III partial scapulectomy [20]. The MUTARS® (Implantcast GmbH, Buxtehude, Germany) glenoid prosthesis and a reverse shoulder prosthesis were used for the reconstruction of glenoid and proximal humerus (Figs. 3, 4). In two patients, due to the extensive involvement of soft tissue, constrained liners were used, to increase the stability of the shoulder and to reduce the possibility of subluxation.

**Fig. 5:**
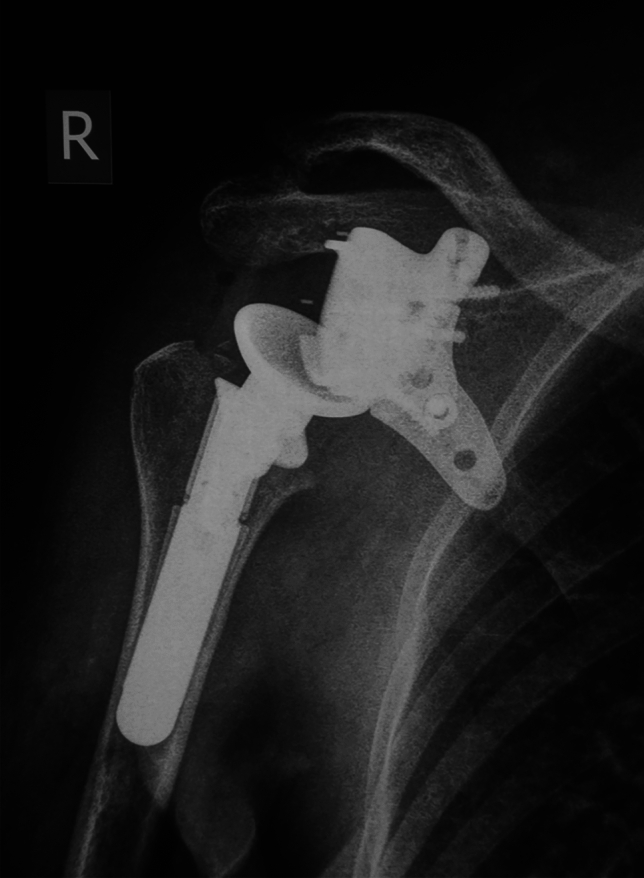
Plain radiograph of the right shoulder 28 months postoperatively

All patients underwent postoperative imaging including plain radiographs and MRI scan to evaluate implant fixation and local recurrence (Fig. 5). Clinical examination was performed to evaluate the postoperative shoulder range of motion (ROM) and the overall functional outcomes. The functional outcomes were assessed through the MSTS (Musculoskeletal Tumor Society Score) and DASH (Disabilities of the Arm Shoulder and Hand) scores [21, 22]. Moreover, patients were asked for their level of satisfaction (very satisfied, satisfied, moderate and poorly satisfied) [23]. Last, the presence of any complications based on the Henderson’s classification system [24] was documented.

Overall, 4 patients were included in this study. There were 2 male patients and 2 females, while the mean age at the time of surgery was 56 (range 48–62) years. Wide resection with negative surgical margins was achieved in all patients according to the pathological examination of the surgical specimens, while the deltoid muscle along with the axillary nerve were preserved in all cases. The mean duration of surgery was 152.5 (range 110–200 min) minutes. The median follow-up period was 22 months (range 12–28 months).

## Results

There were no local recurrences or distant metastases during the follow-up period in any of the included patients. Moreover, there were no postoperative complications based on the Henderson’s Classification System, and no patient required reoperation.

In terms of the functional outcome, the median MSTS score was 80.5% (range 65–94%) and the median DASH score was 15.2% (range 7.5–32.5%). Regarding the postoperative ROM at the latest follow-up, the median active shoulder abduction was 78.75° (range 30–120), and the median active forward flexion was 70° (range 25–110). Three patients were very satisfied with the functional outcome of the procedure, while one was moderately satisfied (Table 1).

**Table 1:** Patients characteristics and functional outcome

Patient	Age	Surgery Time	MSTS	DASH	Abduction	Flexion	Liner
1	48	120	65	32.5	30	25	Constrained
2	56	110	94	7.5	120	110	Conventional
3	58	180	75	11.7	65	45	Constrained
4	62	200	88	9.2	100	100	Conventional

## Discussion

Custom made 3D-printed prostheses have been recently introduced in tumor surgery and offer a patient’s specific alternative reconstructive option. These implants are used to reconstruct complex bone defects following wide excision of musculoskeletal malignancies [25–27]. In our study, customized glenoid endoprosthesis combined with reverse shoulder arthroplasty were used to reconstruct the shoulder girdle and to restore the normal functional of the upper limb after extraarticular Malawer V type resections. The complication rate in our study was low during the short-term follow up, while the range of motion and the functional scores were significantly improved.

Studies regarding glenoid tumors are very limited. Capanna et al. retrospectively reviewed 12 patients who underwent transglenoid resection and reconstruction using prostheses. Although the authors of this study reported that the functional outcomes for this method were better compared to the traditional Tikhoff–Linberg procedure, the overall functional outcome was characterized as fair by most patients, while 25% of the patients underwent hardware removal due to infection [28]. Mnaymneh et al. reported good functional outcomes and a low complication rate using allograft reconstruction after resection of scapula sarcomas. However, in all 6 patients of this study, the rotator cuff muscles were preserved [10]. Similar outcomes were also reported by Zhang et at in 7 patients who underwent reconstruction with allograft [5]. In another study reported by Tsuda evaluated 21 patients who underwent Type V scapular resection followed by reconstruction with proximal humerus endoprosthesis and capture of the prosthesis head to the remaining scapula with either trevira tubes (Implantcast, Germany) or a Mersilene mesh (Ethicon) [29]. The postoperative functional outcomes were poor, while patients were not able to actively elevate their limb. Glenoid tumors are, and although wide excision of these localized lesions can be readily achieved, reconstruction of the residual bone defects is still very challenging [3]. Traditionally, resection of glenoid tumors is associated with poor functional outcomes [5]. The commonly used reconstructive methods include allografts and APCs with comparable results with regard to the functional outcomes and the complications rate. Although these reconstructive methods can result in an acceptable restoration of the upper limb function, they have been associated with a high complication rate (periprosthetic fractures, collapse and non-unions) [2, 3, 10]. Unfortunately, due to the rarity of these tumors, there are no high-quality studies to evaluate whether one reconstructive method is associated with better results compared to another one.

This study has also limitations. This is a retrospective study including a small number of patients with a short-term follow-up. However, due to the rarity of this tumors, there are only few studies evaluating these tumors with similar populations, while our study describes a novel reconstruction method, using 3D-printed implants that seems to offer better functional results.

3D printed, custom made glenoid prosthesis in combination to reverse shoulder arthroplasty can be used in extra-articular, Type V and intraarticular Type III partial scapulectomy excisions of shoulder girdle, with promising outcome. However, studies with larger number of patients and longer follow-up are needed, to establish the results of our study.
